# Downregulation of PGAM2 alleviates angiotensin II-induced cardiac hypertrophy by destabilizing HSP90 and inactivating the mTOR/IKKα signaling pathway

**DOI:** 10.7150/ijbs.102251

**Published:** 2025-01-20

**Authors:** Ying Li, Wen-jing Li, Jia-min Du, Hui-hui Wu, Si-yuan Zhou, Min Li, Yue-yan Li, Shu-ya Wang, Hui-yun Wang, Yan Zheng, Qun-ye Zhang, Li-ming Li, Fan-liang Meng, Guo-hai Su

**Affiliations:** 1 Department of Cardiology, Central Hospital Affiliated to Shandong First Medical University, Jinan, China.; 2 Research Center for Translational Medicine, Jinan Central Hospital, Shandong University, Jinan, China.; 3 The Key Laboratory of Cardiovascular Remodeling and Function Research, Department of Cardiology, Qilu Hospital, Shandong University, Jinan 250012, China.; 4 NingboTech University, Ningbo 315000, Zhejiang, China.; 5 Affiliated Hospital of Shandong University of Traditional Chinese Medicine, Jinan, China.

**Keywords:** Cardiac hypertrophy, PGAM2, HSP90

## Abstract

Pathological cardiac hypertrophy is a major contributor to heart failure. The present study aims to elucidate the role and mechanisms of phosphoglycerate mutase 2 (PGAM2) in the pathogenesis of cardiac hypertrophy. PGAM2 expression was increased in both primary neonatal rat ventricular myocytes (NRVMs) and rat models in response to angiotensin II (Ang II). Downregulation of PGAM2 alleviated cardiac hypertrophy. Mechanistically, we found PGAM2 directly interacts with HSP90 through residues 319-323 and 622-629 in the middle and carboxy-terminal domain of HSP90 respectively. This interaction was further enhanced under Ang II stimulation. Additionally, in the presence of PGAM2, it competed with E3 ubiquitin ligase SYVN1 to interact with HSP90, effectively inhibiting the ubiquitination and degradation of HSP90. Therefore, deficiency of PGAM2 results in the downregulation of the HSP90 and its downstream mTOR and client protein IKKα signaling pathway, both of which play crucial roles in the progression of cardiac hypertrophy. *In vivo*, we further confirmed that PGAM2 knockdown alleviated cardiac hypertrophy through downregulation of HSP90 and mTOR/IKKα signaling pathway. Taken together, we first demonstrated that downregulation of PGAM2 alleviates cardiac hypertrophy induced by Ang II, which provides a novel target for the treatment of myocardial hypertrophy and heart failure.

## 1. Introduction

Heart failure, a late-stage complication of various cardiovascular diseases, is a leading cause of morbidity and mortality worldwide, resulting in millions of deaths and hospitalizations annually. Pathological cardiac hypertrophy is a significant risk factor of heart failure. Recent studies in basic and preclinical research have demonstrated that amelioration of pathological hypertrophy can have a beneficial effect in mitigating the progression of heart failure^1^. Preventing cardiac hypertrophy represents a promising therapeutic approach to the treatment of heart failure. Thus, identifying novel molecules involved in the regulation of cardiac hypertrophy is of great significance for the development of therapeutic interventions.

Phosphoglycerate mutase (PGAM) is a glycolytic enzyme that catalyzes the reversible conversion of 3-phosphoglycerate (3-PG) to 2-phosphoglycerate (2-PG). The human genome contains two PGAM genes: PGAM1, which is expressed in brain and most other tissues, and PGAM2, highly expressed in muscle tissues, especially in skeletal muscle and myocardium^2^. Till now, research of the function of PGAM2 in heart is rare. A previous study demonstrated that persistent overexpression of PGAM2 resulted in systolic dysfunction and reduced stress resistance in the heart upon transverse aortic constriction (TAC)^3^. Additionally, in a proteomic analysis on effluents from perfused human hearts, the concentration of PGAM2 showed an increasing trend when the ischemic time was extending, suggesting its potential as a diagnostic and therapeutic biomarker for myocardial ischemia^4^. Our previous study showed PGAM2 levels are elevated in the serum of heart failure patients and correlate with the disease severity^5^. However, whether PGAM2 plays a role in the regulation of cardiac hypertrophy and the underlying molecular mechanisms remain unclear.

Heat-shock protein 90AA1 (referred to as HSP90 in this work), a member of the heat shock protein family. HSP90 has diverse but selected substrates known as client proteins, and it functions as a key component of the chaperone machinery, assisting in protein folding and modulating the stability and activity of its client proteins^6^. Given its extensive involvement in protein folding and stability regulation, HSP90 has emerged as an attractive therapeutic target to correct various dysfunctions^7^, ^8^. Many client proteins of Hsp90 are involved in pathways known to be pathogenic in the heart, including transforming growth factor β (TGF-β) and mitogen-activated kinase (MAPK) signaling, tumor necrosis factor α (TNFα), Gs and Gq G-protein coupled receptor (GPCR) signaling, and calcium (Ca^2+^) signaling, and also IκB kinase alpha (IKKα) related NFκB signaling^9^, ^10^. mTOR is a key signaling pathways downstream of HSP90, which is a critical regulator in cardiac hypertrophy^11^. Modulation of Hsp90 activity can potentially target these pathways and offer therapeutic opportunities for heart-related pathologies^12^. Recently, HSP90 is proved to play an important role in the regulation of cardiac hypertrophy^13^. HSP90 inhibitors bind to HSP90 and disrupt its association with client proteins, leading to their proteasomal degradation^14^. Inhibitors of HSP90 were proved to attenuate the development of cardiac remodeling and nuclear translocation of p65 NFκB^15^, ^16^. However, most small-molecule inhibitors of HSP90 have been found to be cardiotoxic^10^. Therefore, discovery of regulator of HSP90 is of great significance for modulation of cardiac hypertrophy. Up to now, the endogenous regulator of HSP90 is rarely studied.

In our present study, we clarify that PGAM2 is upregulated in both NRVMs and rat models of Ang II-induced cardiac hypertrophy. PGAM2 deficiency improved the myocardial hypertrophy caused by Ang II. Mechanistically, PGAM2 competed with E3 ubiquitin ligase SYVN1 to interact with HSP90, effectively inhibiting the ubiquitination and degradation of HSP90. Knockdown of PGAM2 led to a dramatic decline in HSP90 protein levels, while HSP90 overexpression reversed the protective effects due to PGAM2 deficiency, implying crucial regulation relationship exists between these two molecules. Collectively, our study identified PGAM2 as a novel regulator of myocardial hypertrophy, suggesting its potential as a therapeutic target for the treatment of heart failure.

## 2. Materials and methods

Detailed materials and methods are available in Supplemental Materials.

## 3. Results

### 3.1 Ang II stimulation leads to upregulation of PGAM2 in NRVMs

**Figure 1:**
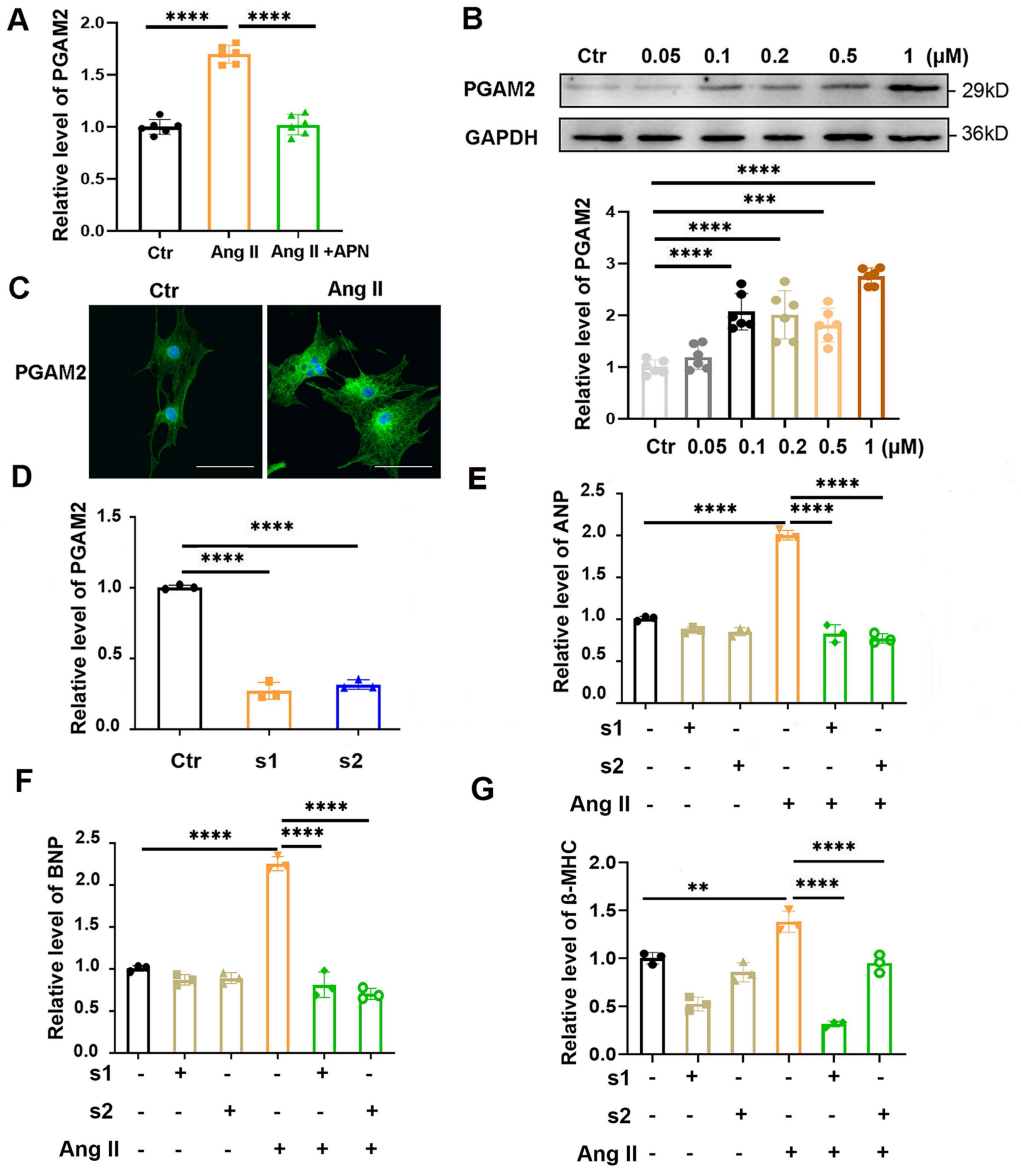
PGAM2 expression, distribution and its effects in Ang II induced cardiomyocyte hypertrophy. (A) The RNA levels of PGAM2 were determined in the control groups, Ang II treatment group and adiponectin (APN) + Ang II group, n = 6. (B) PGAM2 expression was determined by western blot analysis in response to different concentration of Ang II stimulation, n = 6. (C) PGAM2 expression and distribution was detected using Immunofluorescence analysis in cardiomyocytes in the presence or absence of Ang II (Bar = 50 μm). (D) siRNAs targeted PGAM2 were transfected into NRVMs, and the efficiency of RNA interference was determined using qPCR analysis, n = 3. (E-G) Following treatment with Ang II in both the control and PGAM2 knockdown groups, the mRNA levels of ANP, BNP and β-MHC were determined by qPCR analysis, n = 3. One-way ANOVA followed by a post hoc Tukey's test (**, *p* < 0.01. ***, *P* < 0.001. ****, *P* < 0.0001).

In our previous study, we showed that PGAM2 levels are elevated in the serum of heart failure (HF) patients and positively correlated with the severity of HF^5^. However, whether PGAM2 is involved in the regulation of cardiac hypertrophy is unknown. Adiponectin (APN) could alleviate Ang II induced cardiac hypertrophy in our and other studies^17^, ^18^. We observed that the RNA level of PGAM2 was elevated significantly with the stimulation of Ang II, while it was decreased obviously with APN pretreatment in NRVMs (Figure 1A). This changing profile of PGAM2 indicates that it may play a role in Ang II induced cardiac hypertrophy. To further investigate this, we isolated NRVMs from the neonatal rat ventricular and examined the protein level of PGAM2 in response to different concentrations of Ang II stimulation. The identification of NRVMs was presented in Supplementary Figure S1A. We observed a significant increase in PGAM2 protein levels in response to different doses of Ang II (Figure 1B). The immunofluorescence assay revealed that PGAM2 was distributed in both the cytoplasm and the nucleus. Upon stimulation with Ang II, PGAM2 expression was augmented obviously (Figure 1C).

### 3.2 PGAM2 deficiency alleviates Ang II induced cardiomyocytes hypertrophy

To further determine whether PGAM2 is involved in the regulation of Ang II induced cardiac hypertrophy, we conducted experiments to test the cardiac hypertrophy indicators ANP, BNP and β-MHC expression in NRVMs with PGAM2 knockdown or overexpression. As shown in Figure 1D, two siRNAs target against PGAM2 could efficiently decrease the expression of PGAM2. Ang II exposure led to elevated mRNA and protein levels of cardiac hypertrophy indicators ANP, BNP and β-MHC, while PGAM2 silence attenuated these effects (Figure 1E-G, Supplementary Figure S1B-E). However, our results indicated that the overexpression of PGAM2 alone under normal conditions did not effectively induce cardiomyocyte hypertrophy (Figure S2A-D). Therefore, it is only under pathological stimulation of Ang II that elevated PGAM2 levels may mediate the development of cardiomyocytes hypertrophy. Taken together, these results demonstrate that the PGAM2 deficiency may play a protective role in Ang II-induced cardiomyocyte hypertrophy.

### 3.3 PGAM2 directly interacts with HSP90

**Figure 2:**
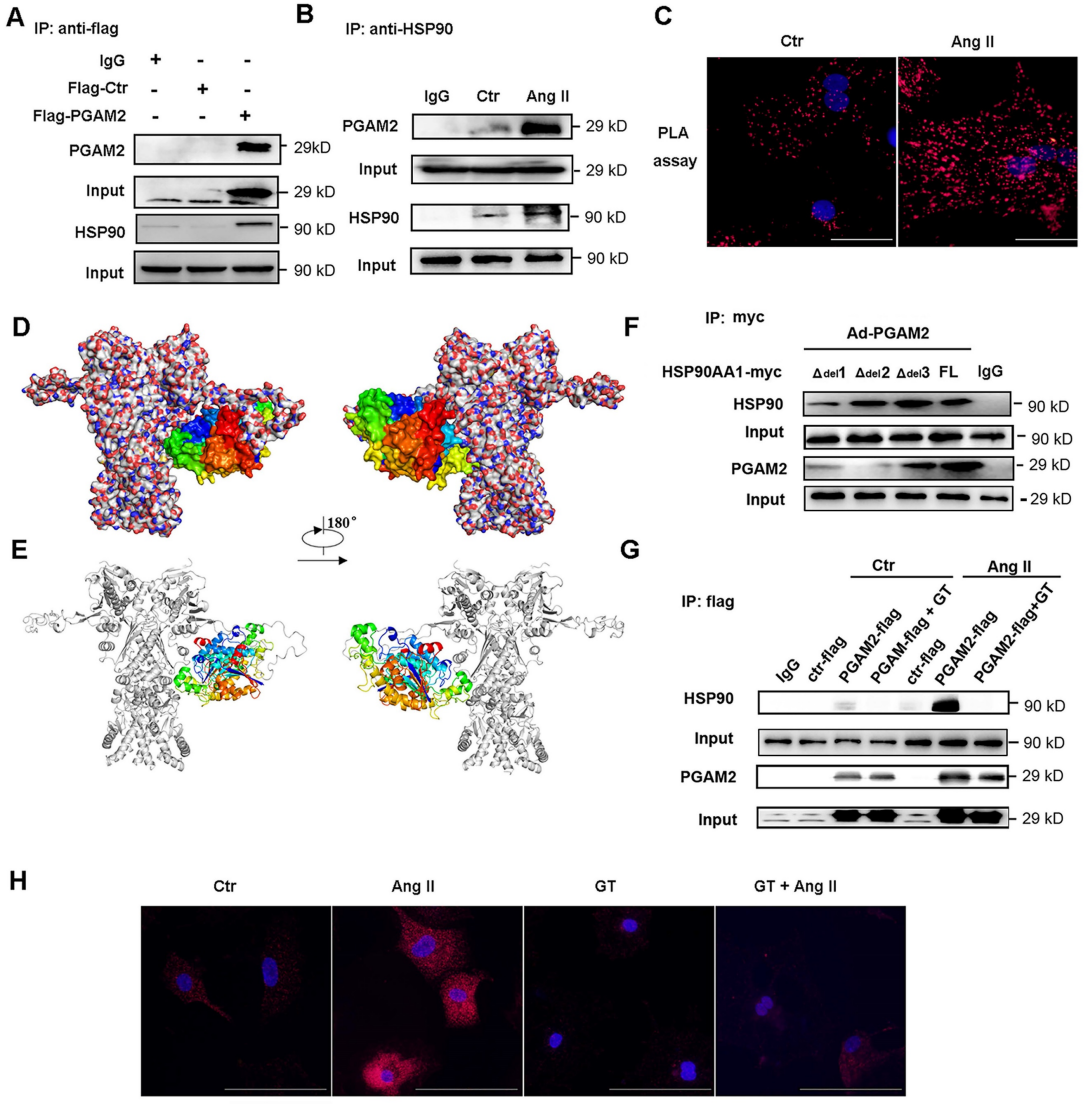
PGAM2 directly interacts with HSP90. (A-B) Co-IP of PGAM2 and HSP90 with anti-flag and HSP90 primary antibodies respectively in NRVMs. (C) The molecular proximity between PGAM2 and HSP90 was analyzed by PLA as described in the Methods. Protein complexes were visualized as red dots in fluorescence. DAPI-stained cell nuclei are shown in blue. Scale bar = 100 μm. (D-E) Predicted overall structure of the PGAM2 and HSP90 complex. Overall representation of the interactive complex structure of PGAM2 and HSP90 depicted with surface mode and cartoon mode. PGAM2 and HSP90 were colored in multicolored and white, respectively. (F) Coimmunoprecipitation experiments with anti-myc antibody were performed in NRVMs co-transfected with overexpression vectors of HSP90 deficient mutants with deletions of residues 622-629 (Δ_del_1), 319-323(Δ_del_2), and 346-351 (Δ_del_3), as well as the full-length HSP90 (FL), and PGAM2 as indicated. n = 3. (G) Coimmunoprecipitation of PGAM2 and HSP90 was determined in NRVMs with or without pretreatment with GT followed by Ang II or PBS (control) treatment. (H) PLA was performed in NRVMs after treatment with GT, Ang II, or a combination of both.

To further investigate the potential molecular mechanism of PGAM2 involved in the regulation of cardiomyocyte hypertrophy, immunoprecipitation (IP) combined with mass spectrometry was performed to detect the interacting protein of PGAM2. HSP90, which is an important molecular chaperone and critical in the regulation of cardiac hypertrophy, was identified as one of the interacting proteins of PGAM2 (Table S1, Figure S3A-J). Ang II stimulation also elevated the protein levels of HSP90 (Figure S4). Based on these findings, we hypothesized that PGAM2 may contribute to the regulation of cardiomyocyte hypertrophy through its interaction with HSP90. To confirm the hypothesis, firstly we verified the interaction between these two proteins. As shown in Figure 2A-B, the results of co-immunoprecipitation (Co-IP) confirmed the binding of these two proteins. Proximity ligation assay (PLA), which is also known as Duolink PLA technology with high sensitivity and specificity to examine the molecular proximity between two proteins, was applied to further detect the interaction between PGAM2 and HSP90 *in situ* at endogenous protein levels. Positive protein interacting complexes were visualized as red dots in fluorescence field images. As shown in Figure 2C, the PLA results confirmed the co-localization of PGAM2 and HSP90. After stimulation with Ang II, red spots augmented and enhanced indicated intensified interaction of PGAM2 and HSP90 compared to the control group.

The obtained protein-protein interactions of PGAM2 with HSP90 were carefully analyzed by homology modelling and molecular docking. The relative interaction complex with the best score was shown in Figure 2D-E. As we can observe from this figure, PGAM2 showed interacting modes with HSP90. HSP90 comprises three critical conserved domains: the amino-terminal domain (NTD, residues 1-220), responsible for ATP binding; the middle domain (MD, residues 255-599) essential for ATP hydrolysis and clients binding; and the carboxy-terminal domain (CTD, residues 599-709), involved in dimerization^19^. Through docking analysis, possible binding sites were identified in residues 319-323 (MD), 346-351(MD), 622-629 (CTD) of HSP90. To further determine the interaction region of PGAM2 and HSP90, we constructed mutant vectors with deletions of residues 622-629 (Δ_del_1), 319-323(Δ_del_2), and 346-351 (Δ_del_3), as well as the full-length HSP90 (FL) adenovirus vectors, which were then transfected into NRVMs. The results showed that deletion of 622-629 (Δ_del_1) and 319-323 (Δ_del_2) significantly weaken the interaction between PGAM2 and HSP90. Notably, deletion of residues 319-323 nearly abolished the binding of PGAM2 and HSP90 (Figure 2F). These findings suggest that the residues 622-629 and 319-323 of HSP90 are required for PGAM2 binding. Deletions in these regions of HSP90 disrupt the interaction with PGAM2.

Ganetespib (GT), also known as STA-9090, was used as a specific inhibitor of HSP90 to investigate its effects on the interaction between PGAM2 and HSP90. GT has been shown to regulate the degradation of several oncogenic HSP90 client proteins^20^. As shown in Figure 2G, Ang II treatment strikingly enhanced the interaction between PGAM2 and HSP90 compared to the control group. However, to our surprise, pretreated NRVMs with GT dramatically blocked this interaction. These results suggest that GT acts as a powerful repressor of the interaction between PGAM2 and HSP90. Additionally, PLA showed consistent results with the Co-IP experiments (Figure 2H), further supporting the finding that GT suppresses the interaction between PGAM2 and HSP90.

### 3.4 PGAM2 maintains the stability of HSP90 and prevents its degradation

**Figure 3:**
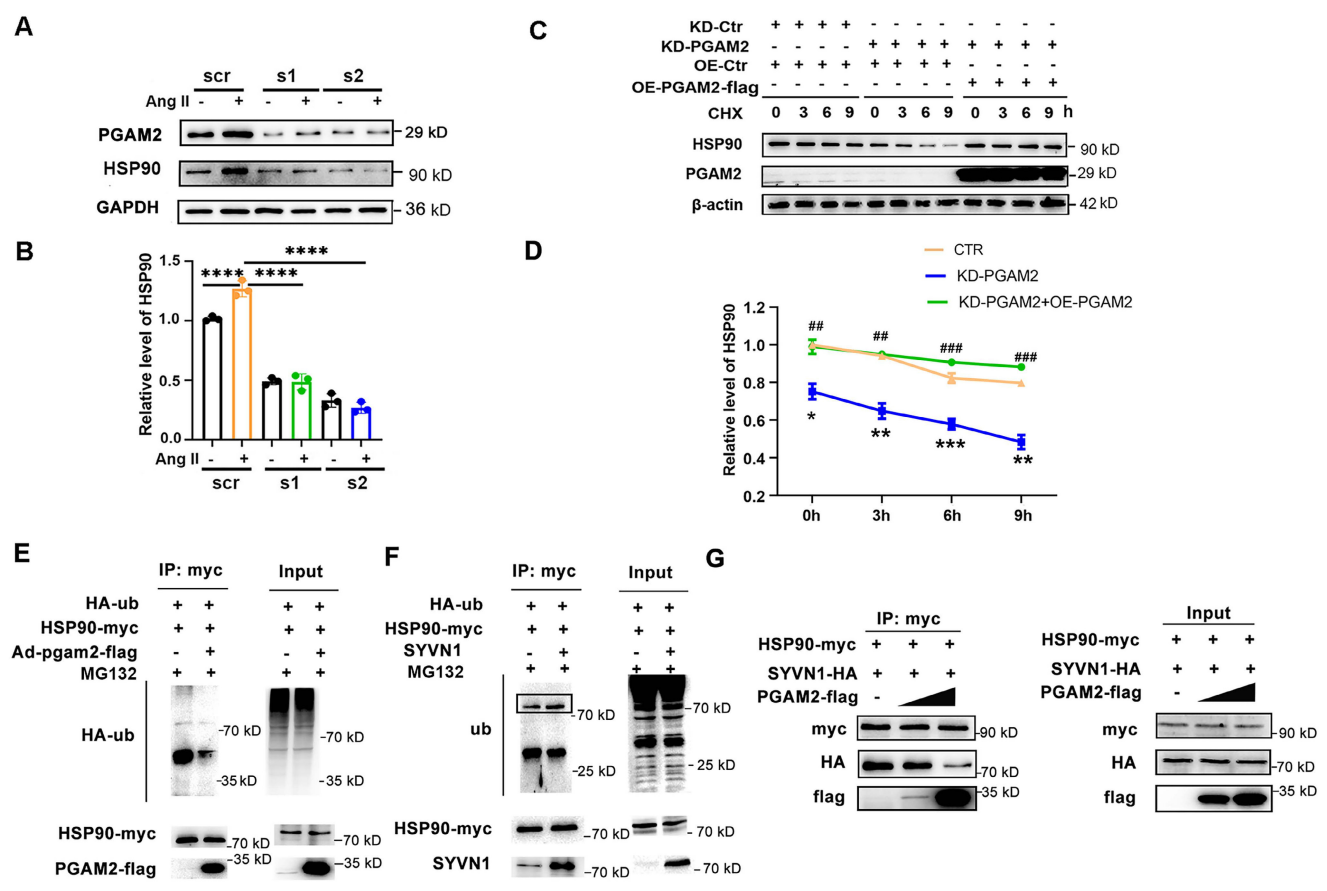
PGAM2 regulates the degradation of HSP90. (A-B) HSP90 protein level was determined by western blotting after transfected with two siRNA (s1 and s2) against PGAM2. n = 3. One-way ANOVA followed by a post hoc Tukey's test. (C-D) Representative western blots and quantification of HSP90 in NRVMs transfected with siPGAM2 (KD-PGAM2) or overexpression adenovirus vector of PGAM2 (OE-PGAM2) followed by treatment with the protein synthesis inhibitor CHX. n = 3. Two-way repeated-measures ANOVA analysis followed by Tukey post hoc tests (*, *P* < 0.05 in KD-PGAM2 vs CTR group. **, *P* < 0.01. ***, *P* < 0.001. #, *P* < 0.05 in KD-PGAM2+OE-PGAM2 group vs KD-PGAM2 group. ##, *P* < 0.01. ###, *P* < 0.001). (E) Lysates from 293T cells transfected with HA-ub, HSP90-myc plasmids, with or without Ad-PGAM2-flag were subjected to IP with myc antibody, followed by SDS-PAGE and western blotting. (F) Lysates from 293T cells transfected with HA-ub, HSP90-myc, with or without SYVN1 plasmids, were subjected to IP with myc antibody, followed by SDS-PAGE and western blotting. (G) Lysates of 293T cells transfected with SYVN1-HA, HSP90-myc, PGAM2-flag plasmids respectively, were co-incubated *in vitro* as indicated, and subjected to immunoprecipitation with myc antibody to determine the interaction of HSP90 with SYVN1 in the presence of varying amounts of PGAM2.

Since HSP90 acts as a crucial molecular chaperone and plays a role in modulating the stability of numerous client proteins, it led us to question whether PGAM2 could be one such client protein and whether it is regulated by HSP90. However, our results showed that HSP90 knockdown had no obvious effects on the protein level of PGAM2 (Figure S5), suggesting that PGAM2 may not be directly regulated by HSP90. The interaction between PGAM2 and HSP90 raises an intriguing possibility: could PGAM2, in turn, have a regulatory role on HSP90? To explore this, we proceeded to investigate the effects of PGAM2 knockdown on HSP90. To our surprise, HSP90 expression was elevated after stimulation with Ang II, but this increase was notably attenuated after PGAM2 knockdown, and this decrease was not due to the changes in HSP90 transcription in PGAM2-silenced NRVMs (Figure 3A-B and Figure S6A). Furthermore, PGAM2 overexpression had no obvious effects on the protein levels of HSP90 (Figure S6B). These findings suggest that PGAM2 regulated HSP90 at the post-transcription level.

To further elucidate the potential molecular mechanism, we examined whether PGAM2 knockdown affects the stability of HSP90. Interestingly, in the presence of the protein synthesis inhibitor cycloheximide (CHX), we observed that silencing PGAM2 resulted in an accelerated degradation and decreased stability of HSP90 compared to the control group, as shown in Figure 3C. However, when re-expressed PGAM2 by transfecting the overexpression adenovirus vector of PGAM2 in PGAM2 knockdown NRVMs, HSP90 levels were restored significantly. These results demonstrated that PGAM2 regulated the protein level of HSP90 through modulating its stability and degradation. We found that pretreatment with proteasome inhibitor MG132 was able to restore HSP90 levels that had decreased by PGAM2 knockdown (Figure S7A). In the presence of PGAM2, the ubiquitination of HSP90 was significantly inhibited in 293T cells that barely expressed PGAM2 (Figure 3E). SYVN1 was predicted as the E3 ubiquitin ligase of HSP90 using online prediction tool ubibrowser (http://ubibrowser.ncpsb.org/ubibrowser/) (Figure S7B). Our results showed that SYVN1 binds with HSP90 and mediated the ubiquitination of HSP90 (Figure 3F). However, in the presence of PGAM2, it competed with SYVN1 to interact with HSP90 (Figure 3G). Taken together, we speculated that PGAM2 effectively inhibits the ubiquitination and degradation of HSP90 through competing with SYVN1 to interact with HSP90.

### 3.5 PGAM2 acts upstream of HSP90 and PGAM2 deficiency ameliorates Ang II-induced cardiomyocyte hypertrophy through downregulation of HSP90

**Figure 4:**
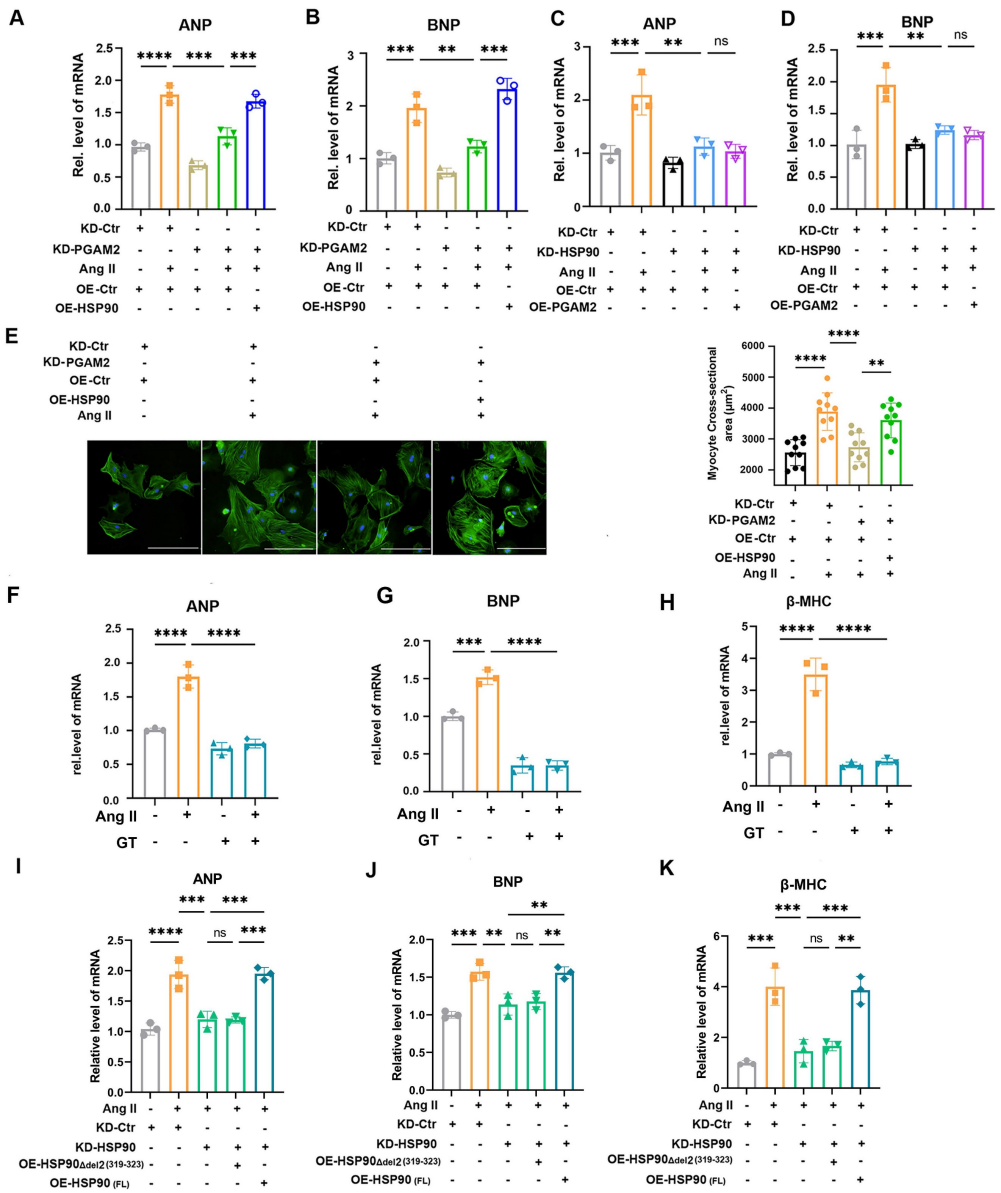
PGAM2 regulates Ang II-induced cardiomyocyte hypertrophy through interaction with HSP90. (A-B) Cardiac hypertrophy indicators ANP and BNP were determined by qPCR in NRVMs with either PGAM2 knockdown or overexpression of HSP90. n = 3. (C-D) ANP and BNP levels detected after HSP90 knockdown or PGAM2 overexpression using qPCR. n = 3. (E) The size of NRVMs were assessed by staining with phalloidin following various treatment. Bar = 200 μm. n = 10. (F-H) NRVMs were pretreated with GT and followed with Ang II stimulation to determine the cardiac hypertrophy markers ANP, BNP and β-MHC. n = 3. (I-K) NRVMs were initially knocked down with HSP90, followed by transfection with either the HSP90 deletion mutant Δdel2 (319-323), or full-length HSP90 vector (FL), and subsequently stimulated with Ang II. qPCR was performed to determine the level of ANP, BNP and β-MHC. n = 3. One-way ANOVA followed by a post hoc Tukey's test (**, *P* < 0.01. ***, *P* < 0.001. ****, *P* < 0.0001. ns, *P* > 0.05).

Next, we determined whether PGAM2 involved in the regulation of cardiomyocyte hypertrophy through HSP90. We showed that silencing PGAM2 decreased the expression of ANP and BNP and β-MHC, while overexpression of HSP90 reversed the protective effects of PGAM2 knockdown in Ang II stimulated NRVMs (Figure 4A-B, Figure S8A). These results provide evidence that PGAM2 participates in the regulation of Ang II-induced cardiomyocyte hypertrophy through HSP90. Consistent with previous reports highlighting the role of HSP90 in modulating cardiac ventricular hypertrophy^12^, we verified that knockdown HSP90 expression by siRNA effectively ameliorated cardiomyocyte hypertrophy (Figure 4C-D and Figure S8B). However, overexpressed PGAM2 with an adenovirus vector was unable to reverse these effects caused by HSP90 knockdown. These results demonstrated that PGAM2 acts upstream of HSP90, regulating its degradation and contributing to the regulation of cardiomyocyte hypertrophy through HSP90. Furthermore, phalloidin staining results further support that PGAM2 knockdown alleviated cardiomyocyte hypertrophy (Figure 4E). In addition, pretreatment with GT, which acts as a strong blocker of the interaction between PGAM2 and HSP90, could attenuate cardiomyocyte hypertrophy remarkably, as evidenced by the decrease expression of ANP, BNP and β-MHC (Figure 4F-H). Consistent with these results, in NRVMs with endogenous HSP90 knockdown and Ang II stimulation, transfection of the HSP90 deletion mutant Δdel2 (319-323) was unable to reverse the protective effect observed with HSP90 knockdown as the full-length HSP90 vector (FL) (Figure 4I-K). Taken together, these findings demonstrated that the interaction of PGAM2 and HSP90 was essential for the stability of HSP90 and finally contributed to the regulation of cardiomyocyte hypertrophy.

### 3.6 PGAM2 regulates cardiomyocyte hypertrophy related to Hsp90 downstream mTOR and client protein IKKα signaling pathway

**Figure 5:**
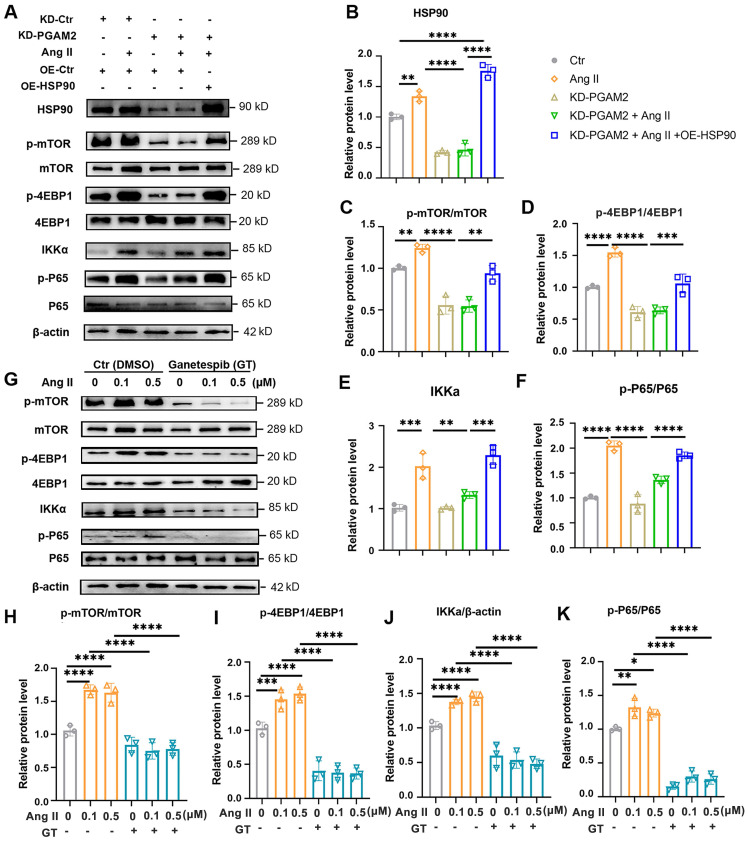
PGAM2 regulates Hsp90 client protein IKKα and mTOR related signaling pathway. (A) Representative western blotting results and (B-F) densitometric quantification of HSP90, p-mTOR/mTOR, p-4EBP1/4EBP1, IKKα, p-P65/P65 in NRVMs with either PGAM2 knockdown or HSP90 overexpression under Ang II stimulation. n =3. (G-K) Representative western blotting results and densitometric quantification of p-mTOR/mTOR, p-4EBP1/4EBP1, IKKα, p-P65/P65 in NRVMs with pretreatment with GT followed by Ang II treatment. n = 3. One-way ANOVA followed by a post hoc Tukey's test (*, *P* < 0.05. **, *P* < 0.01. ***, *P* < 0.001. ****, *P* < 0.0001).

We demonstrated that PGAM2 is involved in the regulation of cardiomyocyte hypertrophy through HSP90, which is a key factor in the progression of cardiac hypertrophy and has numerous client proteins. Herein, we set out to investigate the involvement of PGAM2 in regulating specific HSP90 downstream factor mTOR and its client protein IκB kinase alpha (IKKα), both of which have been shown to be critical in cardiac hypertrophy and heart failure^16^, ^21^. IKKα is involved in the activation of nuclear factor-κB (NFκB), a key regulator required for hypertrophic growth of primary rat neonatal ventricular cardiomyocytes^22^, ^23^. Therefore, to further elucidate the molecular mechanism by which PGAM2 participates in the regulation of cardiac hypertrophy through HSP90, we examined the signaling pathways related to mTOR and IKKα. As shown in Figure 5A-F, stimulation with Ang II led to a significant increase in the phosphorylation levels of mTOR and its downstream substrate 4EBP1, as well as the levels of IKKα/p-p65, indicating the activation of mTOR and NFκB signaling. However, the knockdown of PGAM2 attenuated these effects caused by Ang II. As expect, overexpression of HSP90 restored the activation of mTOR and NFκB signaling pathway. Moreover, pretreatment with GT significantly decreased the levels of p-mTOR/mTOR, p-4EBP1/4EBP1, IKKα, p-p65/p65 levels (Figure 5G-K).

### 3.7 *In vivo* study confirmed PGAM2 deficiency attenuates Ang II-induced cardiac hypertrophy through HSP90

Besides the *in vitro* study, meanwhile, we verified the role of PGAM2 in the regulation of cardiac hypertrophy *in vivo*. Cardiac hypertrophy models were generated by Ang II infusion using subcutaneously implanted osmotic mini-pump for four weeks in wild-type male Wistar rats (200 ng/kg/min). Recombinant AAV-9 vectors carrying shPGAM2 against PGAM2 (AAV9-KD-PGAM2) or OE-HSP90 or the negative control vectors were manufactured and administered to rats via tail vein injection. We confirmed that PGAM2 was efficiently knocked down and HSP90 was successfully over-expressed in rats' heart tissue (Figure S9A-D). Consistent with the results observed *in vitro*, HSP90 expression increased after Ang II exposure but declined noticeably in PGAM2 knockdown group (Figure S9D).

**Figure 6:**
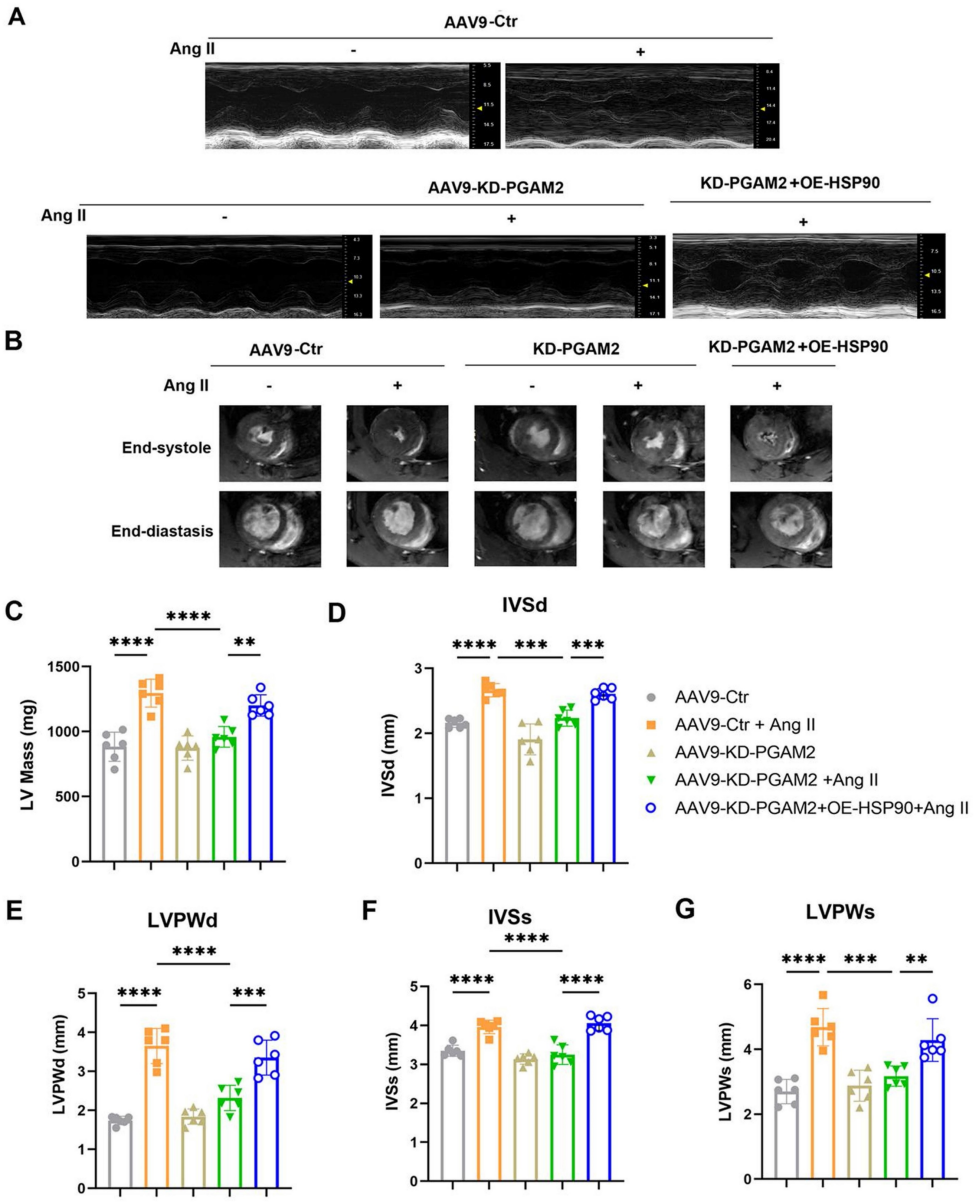
Echocardiography and magnetic resonance imaging (MRI) were performed to determine the ventricular wall thickness in different treatment groups. (A) Representative M-mode echocardiographic images from each group in rats. (B) Representative images of short-axis plan in end-diastole and end-systole hearts by using 9.4 T small animal magnetic resonance imaging (MRI). (C) left ventricular mass of each group was measured. (D-G) The left ventricular end-diastolic interventricular septal thickness (IVSd), end-diastolic posterior wall thickness (LVPWd), interventricular septal thickness at systolic (IVSs), and left ventricular posterior wall at systolic (LVPWs) were quantified with echocardiography. One-way ANOVA followed by a post hoc Tukey's test (**, *P* < 0.01. ***, *P* < 0.001. ****, *P* < 0.0001. n = 6).

**Figure 7:**
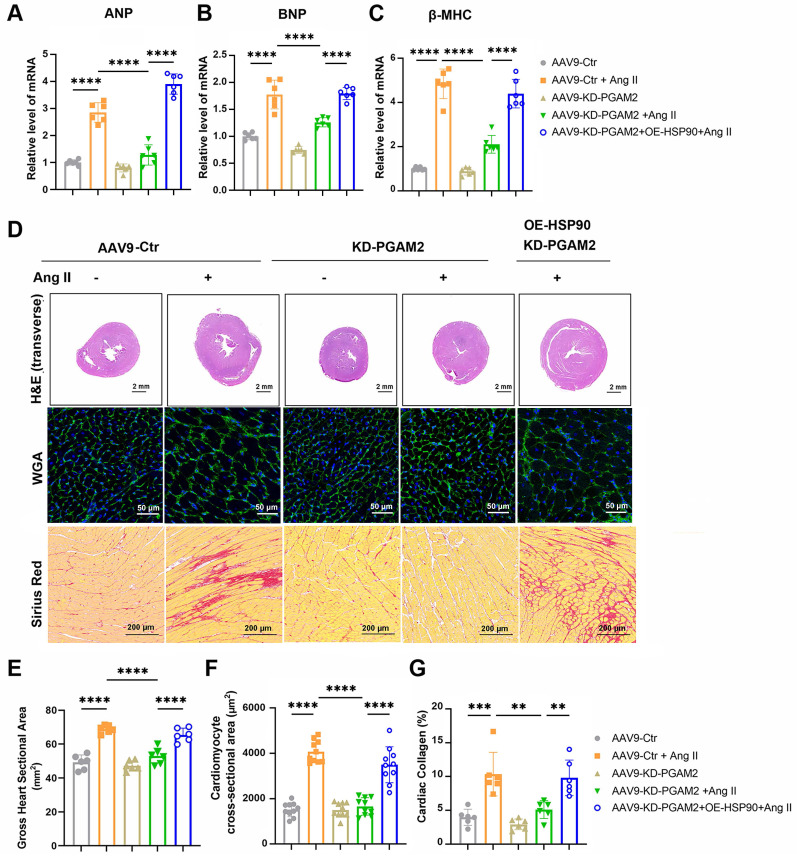
PGAM2 regulates cardiac hypertrophy through HSP90 *in vivo*. (A-C) Expression of cardiac hypertrophy marker genes ANP, BNP, β-MHC detected by qPCR. n = 6. (D-G) Representative images and quantification of Hematoxylin-eosin (H&E), wheat germ agglutinin (WGA), Sirius Red staining in sections of hearts. One-way ANOVA followed by a post hoc Tukey's test. (Scale bar, 2 mm for H&E; 50 μm for WGA; 200 μm for Sirius Red staining. **, *P* < 0.01. ***, *P* < 0.001. ****, *P* < 0.0001. n = 6).

Echocardiography and magnetic resonance imaging results showed that Ang II infusion caused left ventricular hypertrophy compared to the control group of rats, which were manifested as increasing LV mass, thickening of interventricular septal and posterior left ventricular walls (Figure 6A-G). However, ejection fraction and fractional shortening were not significantly altered, indicating the establishment of a cardiac hypertrophy model rather than heart failure (Figure S10A-B). We showed that PGAM2 deficiency ameliorated Ang II induced cardiac hypertrophy as indicated by decreased LV mass, left ventricular walls thickness (Figure 6A-G), as well as reduced expression of ANP, BNP and β-MHC (Figure 7A-C). As expected, HSP90 overexpression reversed these phenomena due to PGAM2 knockdown. Consistently, similar results were observed from H&E and WGA staining (Figure 7D-G). Meanwhile, Sirius red staining results showed aggravated myocardial fibrosis after Ang II stimulation, which was improved by PGAM2 silence. However, these protective effects were canceled when HSP90 was overexpressed in heart tissue.

**Figure 8:**
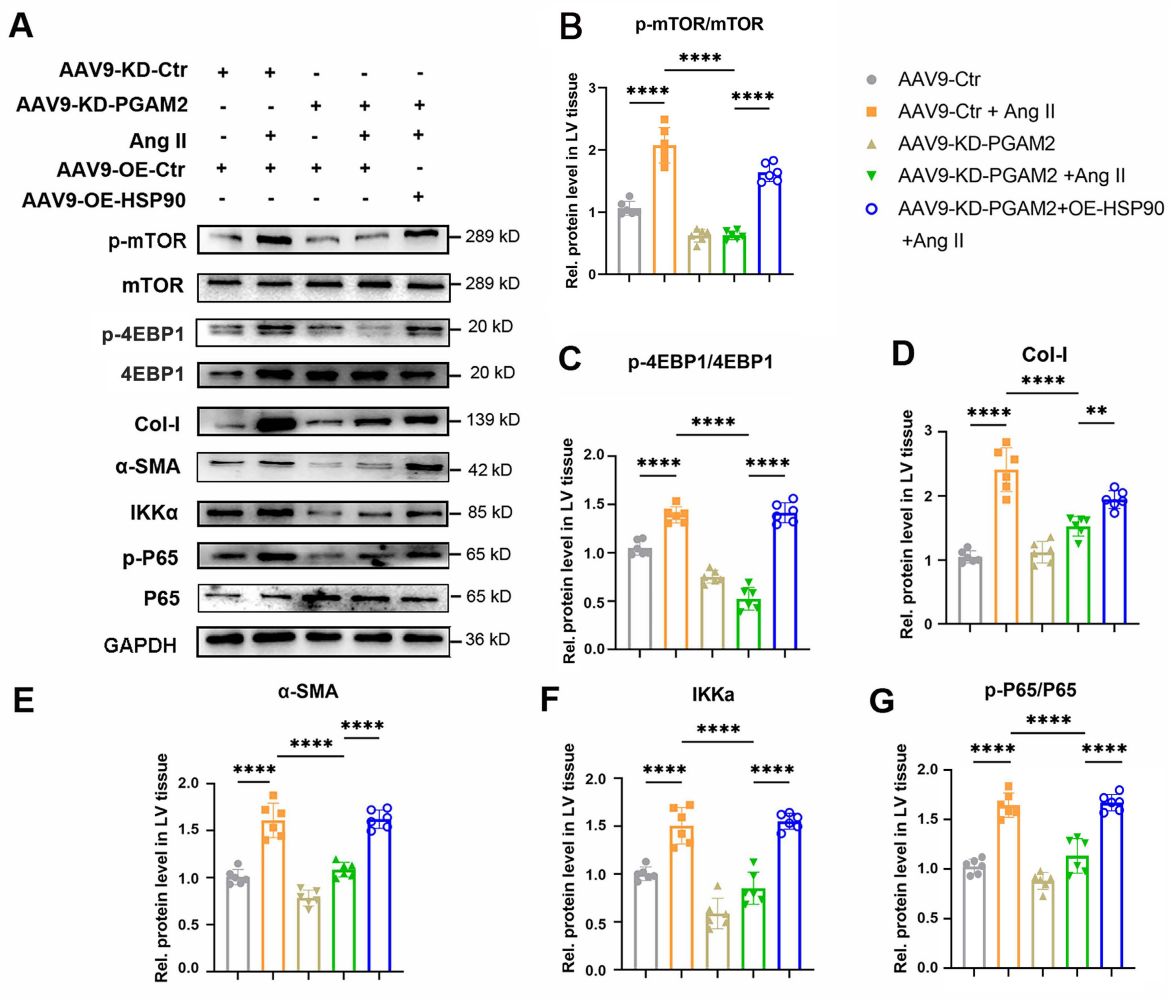
IKKα and mTOR related signaling pathways were detected in cardiac hypertrophy rat models with PGAM2 knockdown or overexpression with AAV-OE-HSP90. (A) Representative western blotting results and (B-G) densitometric quantification of p-mTOR/mTOR, p-4EBP1/4EBP1, Col-I, α-SMA, IKKa, p-65/p65 in left ventricular (LV) tissues. The protein level was standardized by GAPDH. One-way ANOVA followed by a post hoc Tukey's test. (**, *P* < 0.01. ****, *P* < 0.0001. n = 6).

Furthermore, we investigated the expression of HSP90 client proteins IKKα and its downstream mTOR related signaling pathway in different groups of heart tissue. As shown in Figure 8A-G, the mTOR signaling pathway was activated in response to Ang II exposure, as evidenced by elevated phosphorylation levels of p-mTOR and p-4EBP1. Additionally, the expression of IKKα, an essential activator of NFκB, and phosphorylation level of p65 (p - p65) were elevated upon stimulation with Ang II. Nevertheless, when PGAM2 was downregulated, the excessive activation of mTOR and IKKα-related signaling pathways induced by Ang II was suppressed, suggesting a protective mechanism in action. Notably, the introduction of HSP90 overexpression reversed these protective effects, restoring the activation of mTOR and IKKα-related signaling pathways.

## 4. Discussion

In the present study, we demonstrated for the first time that PGAM2 functions as a crucial regulator of cardiac hypertrophy. Our findings from both *in vitro* and *in vivo* experiments showed that the expression of PGAM2 was significantly elevated in models of cardiac hypertrophy induced by Ang II, while PGAM2 knockdown could alleviate Ang II-induced cardiac hypertrophy. It is worth noting that numerous enzymes involved in carbohydrate metabolism, including PGAM2, have been found to exhibit non-enzymatic functions, thus be classified as moonlighting proteins^24^. In addition to its catalytic role in glycolysis, PGAM2 has been implicated in various processes such as tumor progression, lipid accumulation^25^, cell differentiation^25^, ^26^, glycogenosis type X *et al.*^27^. A previous study has suggested that persistent overexpression of PGAM2 leads to systolic dysfunction in response to dobutamine infusion and pressure overload, without affecting the actual glycolytic flux probably. Since the uptake of the glucose and the lactate, the end products of glycolysis, were not changed, with only the metabolites just upstream and downstream of PGAM2 were changed^3^. It was speculated that persistent PGAM2 overexpression may perturb some functions of mitochondria and increase susceptibility to stress. In our previous study, we showed that PGAM2 is elevated in the serum of patients with heart failure and correlates with the disease severity and patient's prognosis^5^. However, whether knockdown PGAM2 level could decrease susceptibility to stress and its involvement in cardiac hypertrophy remains unknown. In the present study, we clarified for the first time that PGAM2 expression was elevated significantly upon the stimulation of Ang II, while PGAM2 deficiency could reverse the cardiac hypertrophy induced by Ang II both *in vitro* and *in vivo*.

By performing IP and mass spectrometry analysis, we identified HSP90 as an interacting protein of PGAM2 that is regulated by PGAM2. We demonstrated that PGAM2 can directly interact with HSP90 via the residue 319-323 in middle domain (MD) and residue 622-629 in the carboxy-terminal domain (CTD) of HSP90. Additionally, PGAM2 may compete with the E3 ligase SYVN1 to interact with HSP90, ultimately effectively inhibiting the ubiquitination and degradation of HSP90. We showed that the deficiency of PGAM2 accelerated the degradation of HSP90 and alleviated Ang II-induced cardiac hypertrophy. While HSP90 overexpression reversed the protective effects of PGAM2 deficiency both *in vitro* and *in vivo*. These results provide evidence for the inter-regulation between PGAM2 and HSP90, deepening our understanding of the mechanism by which PGAM2 is involved in cardiac hypertrophy and degradation process of HSP90. Moreover, our findings revealed that the anti-hypertrophic effects induced by HSP90 knockdown could not be reversed by PGAM2 overexpression, suggesting that PGAM2 functions upstream of HSP90 in this signaling pathway. These results clarify that PGAM2 is involved in the regulation of cardiac hypertrophy through modulation of HSP90, which provides a potential therapeutic target for the treatment of cardiac hypertrophy and HSP90-related dysfunctions.

HSP90 acts as a molecular chaperone involved in the regulation of stability of its client proteins. Numerous client proteins of HSP90 have been identified in known cardiac disease pathways, including PI3K/AKT (PKB)/mTOR, and IKKα signaling, all of which are direct and indirect targets of HSP90^11^. Through interaction and regulation of the stability of HSP90, PGAM2 may play a crucial role in the modulation of its clients. mTOR and IKKα have been proved to be the downstream effector factors of HSP90 and play critical roles in the progression of myocardial hypertrophy^28^. mTOR plays a critical role in the survival of the embryo and the differentiation of cardiac cells^29^. In response to mechanical overload, mTORC1 has been shown to be crucial for the development of cardiac hypertrophy^30^. Partial genetic or pharmacological inhibition of mTORC1 has been observed to reduce cardiac remodeling and the occurrence of heart failure in response to pressure overload and chronic myocardial infarction^31^, ^32^. Several HSP90 inhibitors have been shown to significantly downregulate the mTOR signaling pathway^33^. However, the role of PGAM2 in the regulation of mTOR remains unclear. In the present study, we demonstrated that PGAM2 deficiency leads to a decrease in HSP90, subsequently resulting in the inactivation of mTOR signaling, which is consistent with previous findings. The activation of NFκB has been shown to be essential for hypertrophic growth of NRVMs and is crucial in the progression to heart failure^23^, ^34^, ^35^. Under resting conditions, inactive NFκB dimers (typically p65/p50) are bound to the inhibitor of κB (IκB) in the cytoplasm. Upon stimulation, IκB kinase (IKK)-mediated IκB phosphorylation leads to IκB ubiquitination, facilitating the nuclear translocation and phosphorylation of p65, thereby initiating the transcriptional activity of the NFκB signaling pathway^36^. IKKs can also directly phosphorylate the p65 subunit of NFκB in response to TNFα^37^. As a client of HSP90, the interaction between HSP90 and the IKK complex is necessary for the stability and activity of IKK, thereby activating the downstream NFκB signaling pathway in Ang II-induced cardiac hypertrophy^16^, which is consistent with our results. On another aspect, HSP90 inhibitors bind to HSP90 and disrupt its association with IKK, leading to its proteasomal degradation^38^. However, the relationship between PGAM2 and IKKα/NFκB, as well as the underlying molecular mechanism involved in the regulation of cardiac hypertrophy, has not been reported. In our present study, we demonstrate that PGAM2 is involved in the regulation of mTOR and IKKα/ NFκB signaling through modulation of HSP90 stability, ultimately contributing to Ang II-induced cardiac hypertrophy.

In summary, our study demonstrated for the first time that PGAM2 played important role in regulating pathological cardiac hypertrophy through interacting and modulating HSP90 stability and its downstream mTOR and IKKα / NFκB signaling. These findings extend understanding of the function of PGAM2 in myocardial hypertrophy, indicates that suppression of the elevated levels of PGAM2 may be a promising approach for treating Ang II-induced cardiac hypertrophy and diseases related to HSP90 aberrant regulation. These results may provide a new target for treating heart failure.

## Supplementary Material

Supplementary materials and methods, figures.

## Abbreviations

BafA1: Bafilomycin A1
CTD: carboxy-terminal domain
MD: middle domain
NTD: amino-terminal domain
3-PG: 3-phosphoglycerate
2-PG: 2-phosphoglycerate
NRVMs: neonatal rat ventricular myocytes
PLA: proximity ligation assay
APN: adiponectin
GT: ganetespib
