# Supplementary material for: Downregulation of PGAM2 alleviates angiotensin II-induced cardiac hypertrophy by destabilizing HSP90 and inactivating the mTOR/IKKα signaling pathway
Source: Int J Biol Sci. 2025 Jan 20;21(3):1308–21. doi: 10.7150/ijbs.102251 (PMC11781161; doi:10.7150/ijbs.102251)
Supplement: Supplementary file 1 — Supplementary materials and methods, figures. [file ijbsv21p1308s1.pdf]

1. Supplemental Materials and methods

1.1 Reagents and chemicals

DMEM and bovine calf serum were purchased from Gibco Co. (Carlsbad, CA, USA). Ang II (HY-13948) was obtained from MCE (USA). Antibodies against PGAM2 (SAB2702034) were obtained from Sigma-Aldrich (St Louis, MO, USA). Antibodies against phospho-mTOR (5536S), phospho-4EBP1 (2855S), and phospho-p65 (3033S) were obtained from Cell Signaling Technology (Danvers, MA, USA). Antibodies against HSP90 (13171-1-AP), mTOR (66888-1-Ig), 4EBP1 (60246-1-Ig), GAPDH (60004-1-Ig) and β-actin (66009-1-Ig) were obtained from Proteintech (Wuhan, China). Ganetespib (S1159) was obtained from Selleck (USA). PLA kits were obtained from Sigma-Aldrich (St Louis, MO, USA).

1.2 Ethics statement

In the present study, male Wistar rats weighing 200 ± 10 g and 1- to 3-day-old Wistar rats were used in the current studies. The adult rats were maintained under a 12-hour light/dark cycle in pathogen-free conditions, with unrestricted access to standard rat chow and tap water. All experimental procedures were in accordance with the guidelines from Directive 2010/63/EU of the European Parliament on the protection of animals used for scientific purposes and the NIH Guide for the Care and Use of Laboratory Animals and approved by the Animal Care and Use Committee of Jinan Central Hospital (JNCHIACUC202242).

1.3 Primary culture of neonatal rat ventricular myocytes (NRVMs)

Wistar rats aged 1 to 3 days were anesthetized by inhaling 2% isoflurane for 3 minutes. The ventricles were subsequently minced and digested in phosphate-buffered saline (PBS) supplemented with 200 U of type II collagenase and 0.4% horse serum, undergoing three digestion cycles. Following digestion, the cells were centrifuged and resuspended in Dulbecco's modified Eagle's medium (DMEM) containing 5% fetal bovine serum and 8% horse serum. To remove non-cardiomyocytes, a single preplating step of 1.5 hours was conducted, allowing non-cardiomyocytes to adhere to the bottom of the culture dishes while the unattached myocytes remained in suspension. The unattached myocytes were then plated at a density of 1×10^5 cells/ml in the same medium supplemented with 0.1 mM 5-Bromo-2-deoxyUridine (BrdU). The identity of the NRVMs was confirmed through morphological assessment and immunostaining with an α-actin antibody, revealing that over 95% of the cells were indeed identified as NRVMs.

1.4 Experimental Animals

For lentiviral vector-mediated gene transfer in rat, wild-type male Wistar rats were treated with adeno-associated virus serotype 9 (AAV9) carrying the PGAM2 shRNA knockdown vector, HSP90 overexpression vectors or a negative control virus delivered through the jugular vein two weeks prior to infusion with Ang II. Male Wistar rats were lightly anaesthetized using 3% isoflurane inhalation. Ang II infusion was conducted using subcutaneously implanted osmotic mini-pumps (200 ng/kg/min) for a duration of four weeks. The rats were randomly divided into different groups

based on their treatment with or without Ang II. The control group received sterile saline pumps, with six rats in each group.

1.5 Echocardiography of rat

The rats were lightly anesthetized using 3% isoflurane inhalation. Imaging was performed with the animals positioned in the left lateral decubitus position, utilizing a VisualSonics Vevo 3100LT machine. Images were acquired using M-mode, two-dimensional (2-D), and pulse wave (PW) Doppler techniques. All measurements were calculated by the same observer based on the average of six consecutive cardiac cycles.

1.6 Cardiovascular Magnetic Resonance scans

The rats underwent scanning using 9.4 T small animal magnetic resonance imaging (MRI) (Bruker, BioSpec94/20USR, USA). Briefly, each rat was anesthetized with 3% isoflurane and positioned in a prone orientation within the radiofrequency coil. The chest of each rat was centered in the coil, electrodes were inserted into the forelimb and right hindlimb, and a respiration sensor was affixed to the abdomen. MRI scans were conducted once the respiration and electrocardiogram (ECG) signals stabilized. A three-plane localization sequence scan was performed on the chest, followed by the acquisition of four-chamber, two-chamber, and short-axis images of the heart based on the localization image. The parameters were as follows: echo time (TE) of 2 ms, repetition time (TR) of 8.00 ms, flip angle (FA) of 15°, slice thickness of 1.5 mm, field of view (FOV) of 40 mm × 40 mm, with a matrix size of 192×192.

The number of excitations (NEX) was set to four, and 15 film images were collected per cardiac cycle. The total scan duration was 30 to 40 minutes. Global cardiac function was assessed by analyzing the cine images using cvi42 software (Circle, Calgary, Alberta, Canada).

1.7 Hematoxylin and eosin (H&E) staining

Myocardial tissues were sectioned in a cross-sectional orientation, fixed in a 4% paraformaldehyde solution, and subsequently embedded in paraffin for tissue sectioning. Hematoxylin and eosin (H&E) staining was performed on the tissue sections. Images were captured using a bright field microscope (Olympus BX53) and analyzed for cell size using Image Pro Plus 7.0 software. The data presented represent analyses from six independent experiments.

1.8 Immunofluorescence staining

For immunofluorescence staining, cells from each experimental group were cultured on coverslips and subjected to various treatments as indicated. Subsequently, the cells were fixed in 4% paraformaldehyde for 15 min at room temperature, and permeabilized with 0.3% Triton/PBS for three min. After blocking with 10% donkey serum for 45 min, cells were incubated overnight at 4 °C with rabbit anti-PGAM2 antibody (dilution 1:200). Then, cells in each group were incubated with Alexa Fluor 488 conjugated donkey Anti-Rabbit IgG secondary antibody for one hour. Finally, the cells were incubated with DAPI-containing mounting media and observed using a

fluorescence microscope (Olympus, Japan). The immunofluorescence images were quantified using Image J software.

1.9 Analysis of mRNA expression by real-time PCR

Total RNA was extracted using TRIzol reagent (Invitrogen) in accordance with the manufacturer's instructions. cDNA synthesis was conducted using the PrimeScript RT reagent kit with gDNA Eraser (Takara), utilizing 1 μg of RNA. Real-time PCR amplification reactions were performed in triplicate with the SYBR Premix Ex Taq kit containing ROX (Takara) on an ABI Prism 7900 Real-Time PCR machine. The expression levels of the gene of interest were quantified using the ΔΔCT method and normalized against β-actin mRNA levels. The results are presented as fold changes in gene expression relative to the control groups. The primer sequences used for PCR amplification were as follows: PGAM2 forward, 5’-GTCCTTATTGCAGCCCATGG-3’ and reverse, 5’-ACCCACTCTCACTTTGCCTT-3’; atrial natriuretic peptide (ANP) forward, 5-GGGGGTAGGATTGACAGGAT-3 and reverse, 5-GGATCTTTTGCGATCTGCTC-3; and brain natriuretic peptide (BNP) forward, 5-GCTGCTTTGGGCAGAAGATA -3’ and reverse, 5-GGAGTCTGCAGCCAGGAGGT -3; β -myosin heavy chain (β-MHC) forward, 5'-CGCTCAGTCATGGCGGAT-3' and reverse, 5'-GCCCCAAATGCAGCCAT- 3’; Hsp90 forward, 5'-CCAAGGACCAGGTTGCTAACTCA-3' and reverse, 5'-GACACCAAGGTCTTGCCCTCA- 3’; β-actin forward, 5-CGTTGACATCCGTAAAGACC -3 and reverse, 5-

TAGAGCCACCAATCCACACA -3. All real-time experiments have been repeated three times to ensure reproducibility and accuracy of the results.

1.10 RNA interference (RNAi)

NRVMs were transfected with siRNA targeting PGAM2 or HSP90, or with scrambled siRNA at a concentration of 50 nM for 24 h using lipofectamine 2000, following the manufacturer's protocol (Invitrogen, USA). The effciency of RNAi was determined by Western blotting or qPCR analysis.

1.11 Co-Immunoprecipitation (Co-IP)

After various treatments, cells were lysed in ice-cold immunoprecipitation lysis/wash buffer (100 μL/1 $\times 10^6$ cells, Servicebio) at 4 °C for 30 min, followed by centrifugation at 12,000 rpm for 10 minutes. An equal amount of cell lysates (200 μL) was incubated overnight at 4 °C with anti-flag, anti-HSP90, or IgG antibody, along with A/G agarose (Beyotime, 20 μL). Following incubation, the samples were centrifuged at 1000 g for 10 minutes, and the supernatant was discarded. The immunoprecipitated complexes were washed three times with lysis buffer, and then eluted in sample buffer containing 1×sodium dodecyl sulfate (SDS) loading buffer by boiling for SDS-PAGE. Subsequently, the complexes were subjected to immunoblot analysis to detect the interacting proteins.

1.12 LC-MS/MS Analysis

After transfected with flag-PGAM2 and flag-Ctr vectors, NRVMs were lysed in ice-cold immunoprecipitation lysis/wash buffer. Flag antibody was added to the

samples for IP experiment. Then, the LC-MS/MS analysis was carried out by PTM Bio Co., Ltd (Zhejiang, China). Finally, we screened out the proteins that could bind to PGAM2 according to the score and the mass of detected proteins.

1.13 Western blot analysis

Cells were lysed in a protein lysis buffer containing 1% SDS, 25 mM Tris–HCl (pH 7.5), 100 mM NaCl, 4 mM EDTA, 1 mM PMSF, 10 mg/mL leupeptin, and 10 mg/mL soybean trypsin inhibitor. The protein concentration of the lysates was determined using the Coomassie Brilliant Blue protein assay. Subsequently, protein extracts from NRVMs (10 μg) were loaded onto 12% or 6% SDS polyacrylamide gels for electrophoresis and then transferred to a PVDF membrane. Following the transfer, the membranes were incubated with the appropriate primary antibodies to detect the proteins of interest. The target proteins were detected with a horseradish peroxidase-conjugated IgG secondary antibody. Band intensity was quantified using ImageJ software (Bio-Rad, USA) and normalized to internal control levels.

1.14 Proximity ligation assay (PLA)

Duolink In Situ reagents from Olink Bioscience enable detection of protein interactions in tissue and cell samples prepared for microscopy. The Duolink In Situ reagents utilize in situ PLA, a proximity ligation assay technology. This assay allows for the detection of the interaction between PGAM2 and HSP90 using two primary antibodies. To visualize the interaction, a pair of oligonucleotide-labeled secondary antibodies (known as PLA probes, one PLUS and one MINUS) is utilized. These PLA

probes generate a signal only when they bind in proximity, with two primary antibodies that have bound to the sample in proximity. The signal from each detected pair of PLA probes is visualized as an individual fluorescent spot. These PLA signals can be quantified (counted) and assigned to a specific subcellular location based on microscopy images. All the procedures were performed according to the manufacturer's instructions.

1.15 Homology modelling and molecular docking

Homology modelling was conducted using the online program SWISS MODEL (http://swissmodel.expasy.org/). Initially, the primary amino acid sequence of HSP90 from the rat was uploaded to the program. The SWISS-MODEL template library (SMTL version 2020-04-08, PDB release 2020-04-03) was then searched using BLAST and HHBlits to identify evolutionary related structures that match the target sequence. Based on the results obtained, a suitable template was selected for the homology modeling of the HSP90 crystal structure. The model was constructed using ProMod3, which aligns the target sequence with the chosen template. Conserved coordinates between the target and the template were copied from the template to the model, while insertions and deletions were remodeled using a fragment library. Side chains are subsequently rebuilt, and the geometry of the resulting model was optimized using a force field. If loop modeling with ProMod3 was unsuccessful, an alternative model was generated using PROMOD-II. The global and per-residue model quality was assessed using the QMEAN scoring function. The constructed model was selected for subsequent molecular docking simulation. Similar procedures

were employed for the homology modeling simulation of the full length PGAM2 (Rat organism) and HSP90 (Rat organism). The templates for PGAM2 and HSP90 were 6H26 and 5FWM, respectively.

1.16 Protein-protein docking simulation

The online Z-DOCK server (http://zdock.umassmed.edu/) was utilized to perform the protein-protein docking simulaitons. In the simulation of HSP90 with PGAM2, the modeled HSP90 (dimer) and PGAM2 (dimer) were uploaded as Input Protein 1 and Input Protein 2 respectively. The ZDOCK 3.0.2f + IRaPPA re-ranking ZDOCK version was selected for the simulation, with no interacting residues defined. For the simulation, the top-ranking 10 predicted protein-protein interaction modes were retrieved for further evaluation.

1.17 Statistical Analysis

The data are presented as mean ± standard deviation (SD) and were analyzed using GraphPad Prism 9.0 software (San Diego, CA, USA). To determine significant differences, a two-tailed Student's t-test was employed for comparisons between two groups, while one-way ANOVA followed by Tukey's post-hoc test was utilized for comparisons involving more than two groups. Figures were processed using Adobe Photoshop software. For samples requiring repeated measurements from a single individual, independent analyses of the data sets were conducted using two-way repeated-measures ANOVA. This analysis employed a single pooled variance and applied the Tukey correction for pairwise comparisons within groups for each data set. The exact group size (n) for each experiment is provided, and 'n' refers to biological

replicates rather than technical replicates. Differences with a $p < 0.05$ were considered statistically significant.

2. Supplementary Table S1

MS spectra peptide sequences and scores of HSP90 from PGAM2-IP samples.

Sequence	Protein	Score
APFDLFENR	HSP90	95.815
ELHINLIPNK	HSP90	71.349
ELISNSSDALDK	HSP90	73.885
ESDDKPEIEDVGSDEEEEEK	HSP90	54.834
HIYFITGETK	HSP90	126.24
HLEINPDHSIIETLR	HSP90	68.944
LGIHEDSQNR	HSP90	99.136
NPDDITNEEYGEFYK	HSP90	185.25
YYTSASGDEMVSLK	HSP90	117.09

Single-letter abbreviations for the amino acid residues are: A, Ala; D, Asp; E, Glu; F, Phe; G, Gly; H, His; I, Ile; K, Lys; L, Leu; M, Met; N, Asn; P, Pro; Q, Gln; R, Arg; S,Ser; T, Thr; V, Val; W, Trp; Y, Tyr.

3. Supplementary Figures

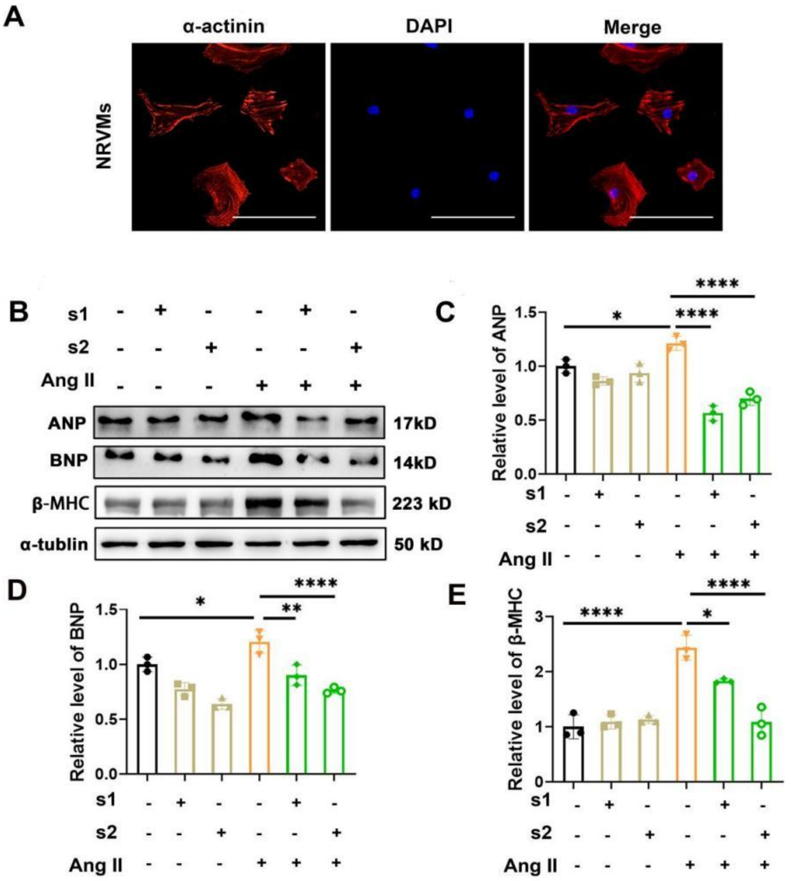

Supplementary Figure S1. Identification of NRVMs and cardiac hypertrophy indicators ANP, BNP and β-MHC expression after different treatments. (A) Immunofluorescence was performed for the identification of NRVMs. Bar = 50μm. (B-E) Following treatment with Ang II in both the control and PGAM2 knockdown groups, the protein levels of ANP, BNP and β-MHC were determined by western blot (*, $P < 0.05$. **, $P < 0.01$. ****, $P < 0.0001$. n = 3).

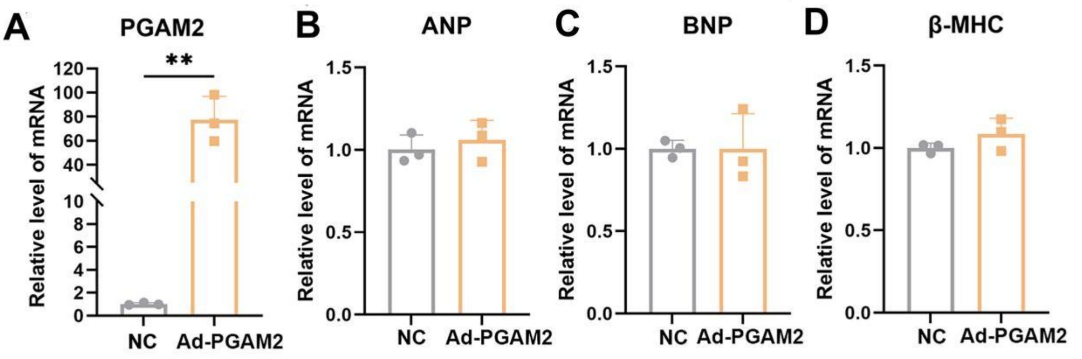

Supplementary Figure S2. After transfected with Ad-PGAM2 in NRVMs, the relative mRNA levels of (A) PGAM2, (B) ANP, (C) BNP and (D) β-MHC were

determined by qPCR. n = 3 (**, $p < 0.01$).

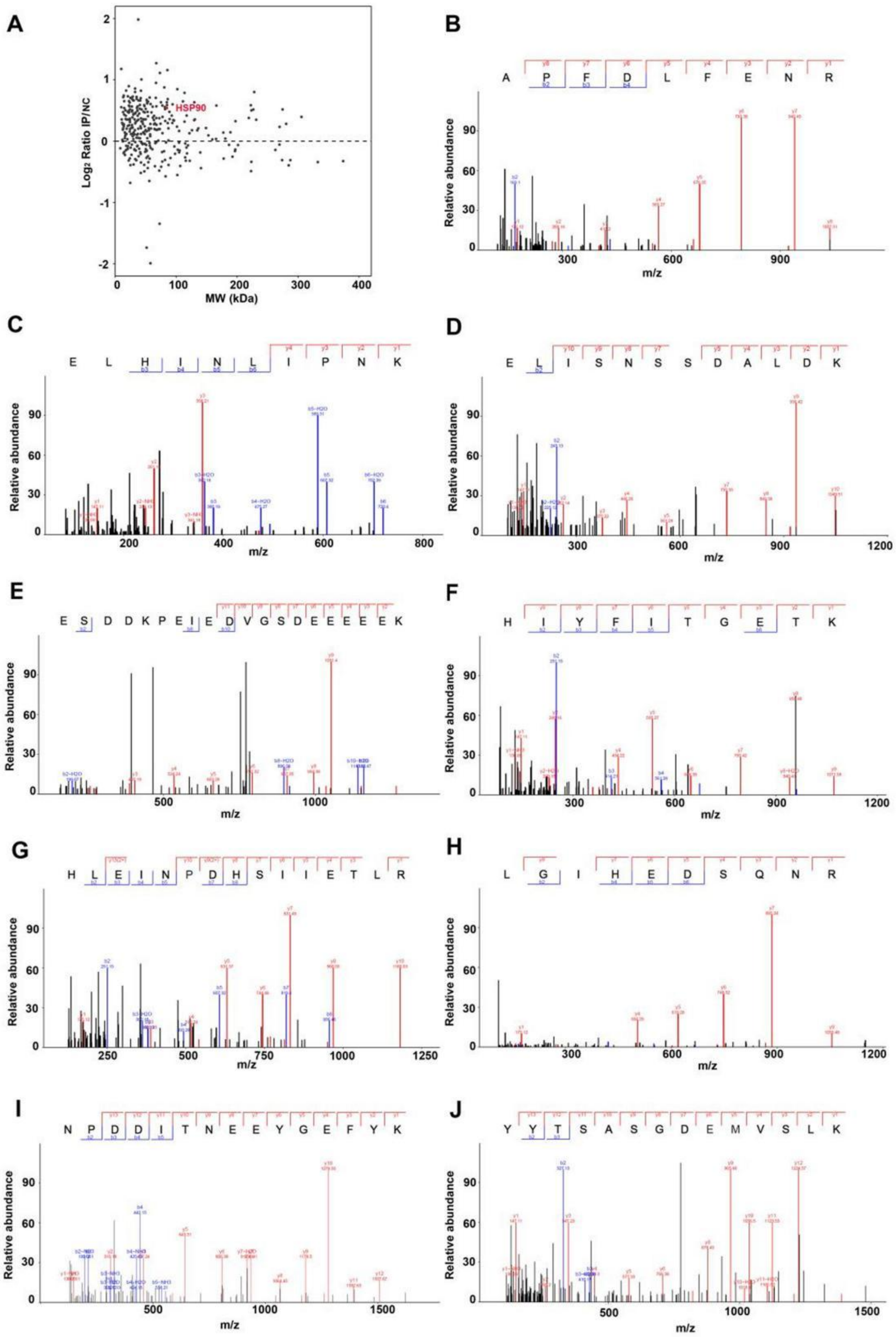

Supplementary Figure S3. (A) Two-dimensional (2D) plot with $\log_2$ IP/NC ($\log_2$ PGAM2-IP score/NC-IP score ratios) of the quantified proteins. The y-axis represents the enrichment in flag-PGAM2 immunoprecipitation (PGAM2-IP) versus flag-Ctr immunoprecipitation (NC-IP), while the x-axis represents the molecular weight (MW) of the protein. (B-J) Mass spectrometry (MS) spectra peptides confirming the

interaction between PGAM2 and HSP90. The identified peptides from HSP90 include APFDLFENR (B), ELHINLIPNK (C), ELISNSSDALDK (D), ESDDKPEIEDVGSDEEEEEK (E), HIYFITGETK (F), HLEINPDHSIIETLR (G), LGIHEDSQNR (H), NPDDITNEEYGEFYK (I) and YYTSASGDEMVSLK (J). The single-letter abbreviations for the amino acid residues are as follows: A, Ala; D, Asp; E, Glu; F, Phe; G, Gly; H, His; I, Ile; K, Lys; L, Leu; M, Met; N, Asn; P, Pro; Q, Gln; R, Arg; S,Ser; T, Thr; V, Val; W, Trp; Y, Tyr. m/z, represents mass/charge ratio.

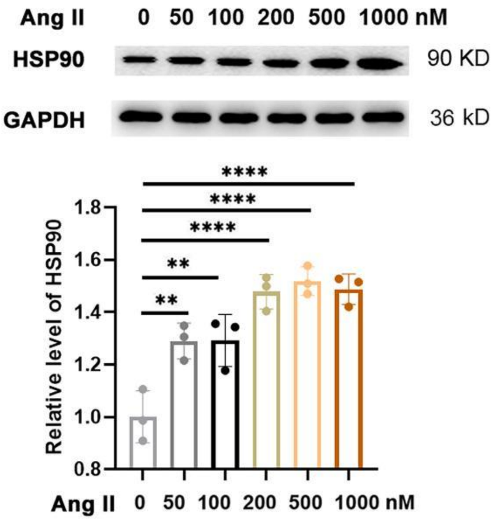

Supplementary Figure S4. Representative western blotting for HSP90 under stimulation with different doses of Ang II and densitometric quantification (**, *P* < 0.01. ****, *P* < 0.0001. n = 3).

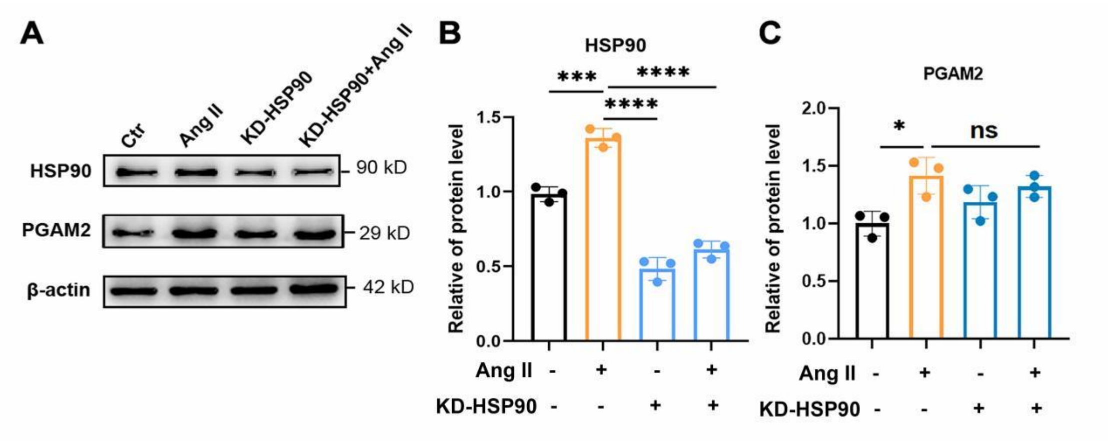

Supplementary Figure S5. The expression of HSP90 and PGAM2 in HSP90

knockdown NRVMs followed by Ang II treatment. (A) Representative western blotting for HSP90 and PGAM2 under different stimulation and (B-C) densitometric quantification (*, $P < 0.05$. ***, $P < 0.001$. ****, $P < 0.0001$. ns, $P > 0.05$. n = 3).

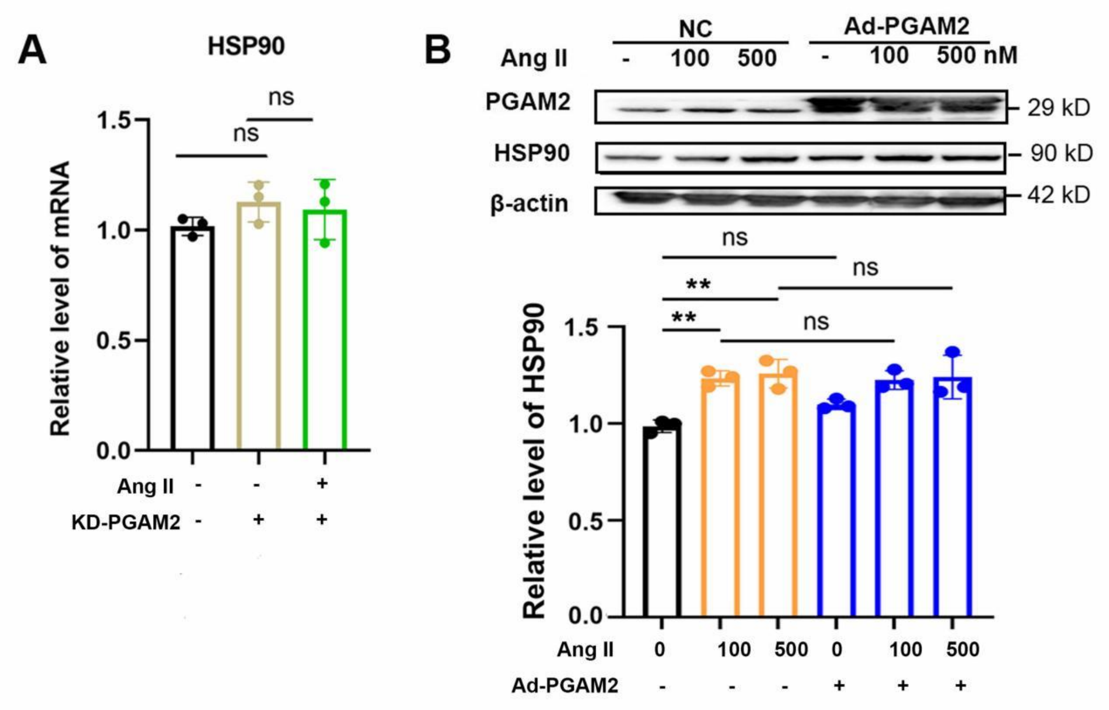

Supplementary Figure S6. HSP90 expression in NRVMs with PGAM2 knockdown (KD-PGAM2) or overexpression (Ad-PGAM2). (A) qPCR analysis of the mRNA levels of HSP90 in NRVMs with KD-PGAM2 followed by Ang II stimulation. (B) Representative western blotting for HSP90 and PGAM2 in Ad-PGAM2 NRVMs and densitometric quantification for HSP90 (**, $P < 0.01$. ns, $P > 0.05$. n = 3).

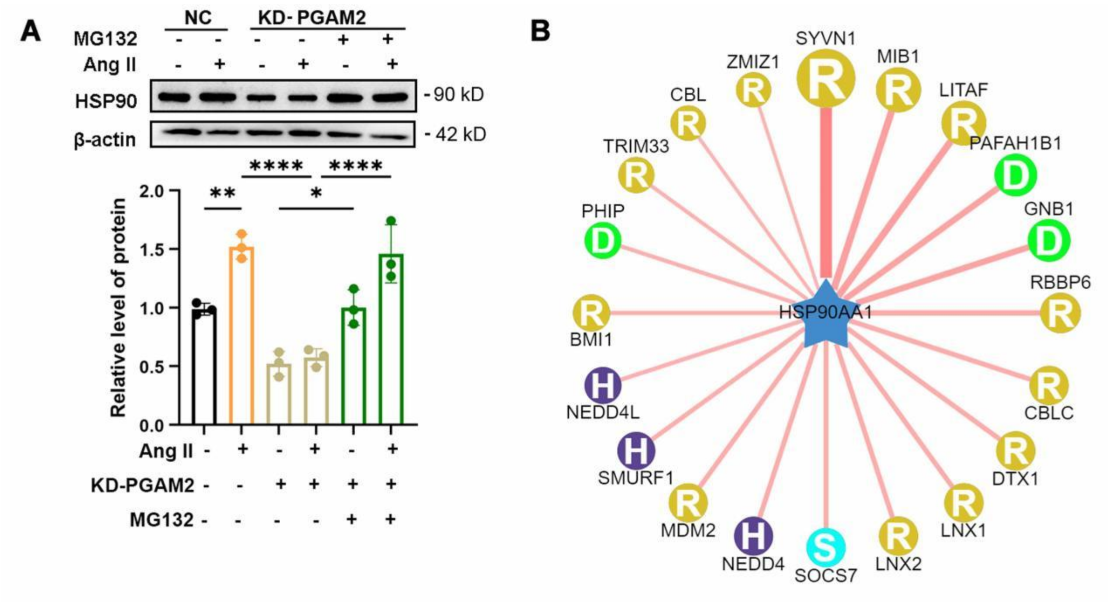

Supplementary Figure S7. The effects of MG132 on the expression of HSP90 under various treatments. (A) Representative western blotting for HSP90 and densitometric quantification (*, $P < 0.05$. **, $P < 0.01$. ****, $P < 0.0001$. n = 3). (B) Prediction of the E3 ubiquitin ligase of HSP90 through online prediction tool ubibrowser (http://ubibrowser.ncpsb.org/ubibrowser/).

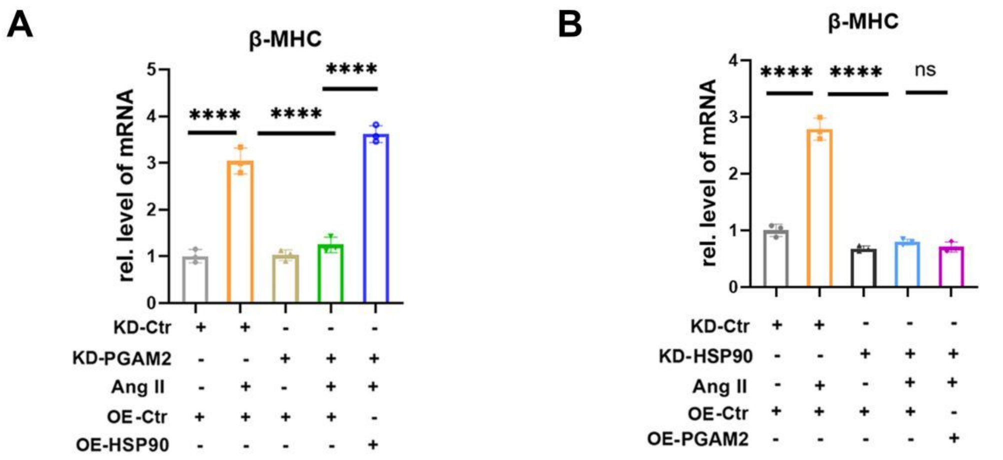

Supplementary Figure S8. The mRNA levels of β-MHC after different treatment. (A) Cardiac hypertrophy indicators β-MHC levels were determined by qPCR in NRVMs with either PGAM2 knockdown or overexpression of HSP90. n = 3. (B) β-MHC levels were detected after HSP90 knockdown or PGAM2 overexpression using qPCR (****, $P < 0.0001$. ns, $P > 0.05$. n = 3).

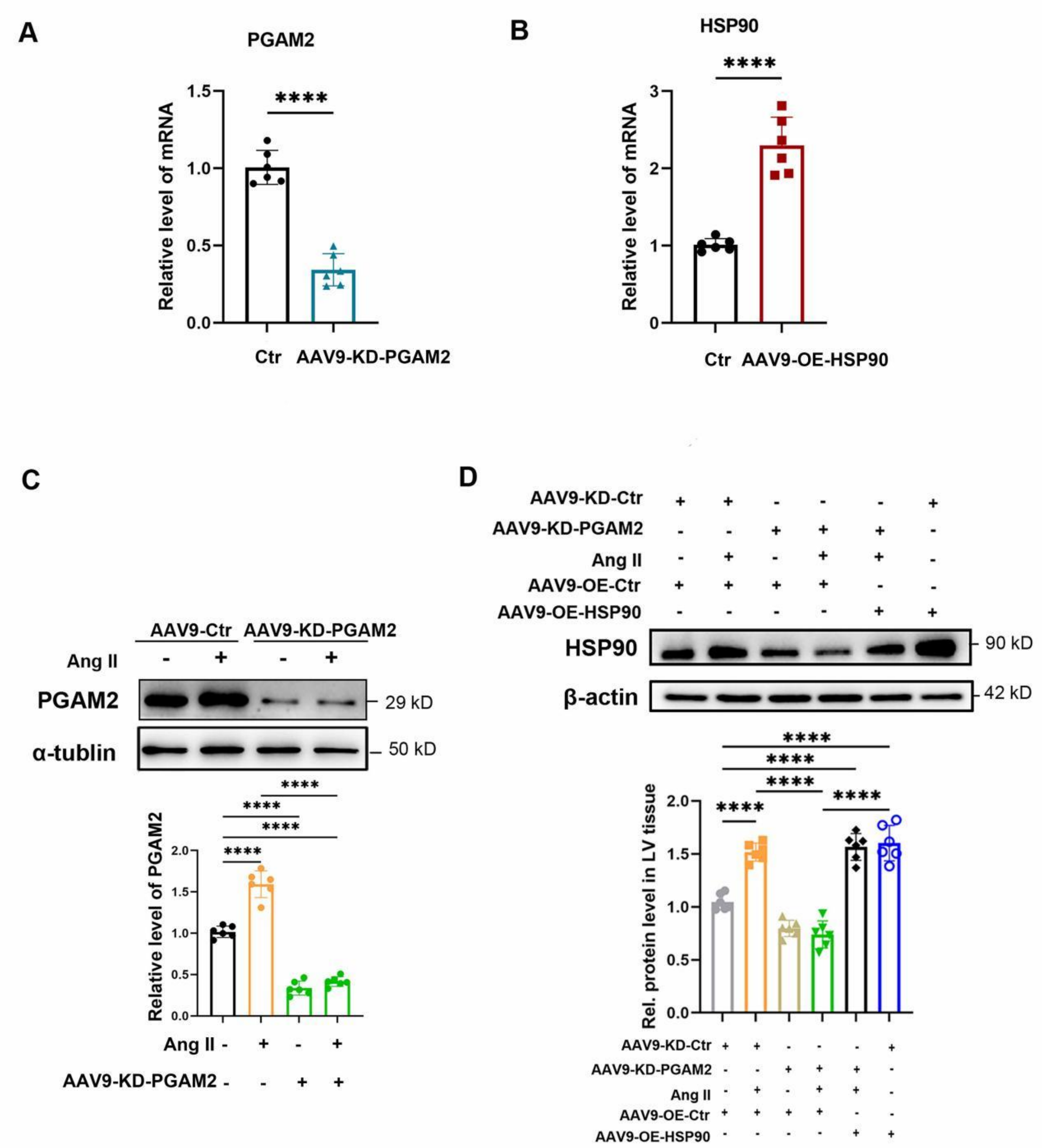

Supplementary Figure S9. The efficiency of PGAM2 knockdown and HSP90 overexpression in heart tissue were detected by qPCR and western blotting. (A) RNAi efficiency of PGAM2 detected by qPCR, n = 6; (B) The over-expression efficiency of HSP90 was detected by qPCR, n = 6. Representative western blotting for (C) PGAM2 and (D) HSP90 under various stimulation conditions, n = 6. (****, $P < 0.0001$).

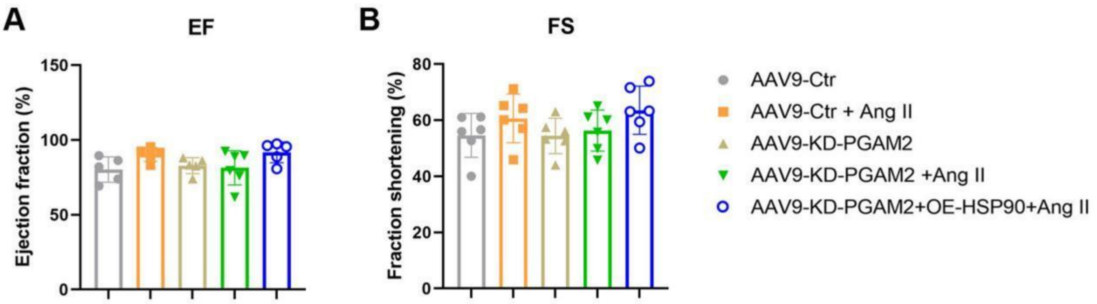

Supplementary Figure S10. (A) EF and (B) FS were quantified with echocardiography in different groups.
